# Fomite-mediated transmission as a sufficient pathway: a comparative analysis across three viral pathogens

**DOI:** 10.1186/s12879-018-3425-x

**Published:** 2018-10-29

**Authors:** Alicia N.M. Kraay, Michael A.L. Hayashi, Nancy Hernandez-Ceron, Ian H. Spicknall, Marisa C. Eisenberg, Rafael Meza, Joseph N.S. Eisenberg

**Affiliations:** 1 Department of Epidemiology, University of Michigan, 1415 Washington Heights, Ann Arbor, MI USA; 2 Department of Mathematics and Statistics, University of Alberta, 632 Central Academic Building, Edmonton, T6G 2G1 AB Canada; 3 Division of STD Prevention, Center for Disease Control, 1600 Clifton Rd. NE Mail Stop E-80, Atlanta, 30333 GA USA

**Keywords:** Environmental Infection Transmission System (EITS), Fomite mediated transmission, SIR epidemic model, Mathematical model

## Abstract

**Background:**

Fomite mediated transmission can be an important pathway causing significant disease transmission in number of settings such as schools, daycare centers, and long-term care facilities. The importance of these pathways relative to other transmission pathways such as direct person-person or airborne will depend on the characteristics of the particular pathogen and the venue in which transmission occurs. Here we analyze fomite mediated transmission through a comparative analysis across multiple pathogens and venues.

**Methods:**

We developed and analyzed a compartmental model that explicitly accounts for fomite transmission by including pathogen transfer between hands and surfaces. We consider two sub-types of fomite-mediated transmission: direct fomite (e.g., shedding onto fomites) and hand-fomite (e.g., shedding onto hands and then contacting fomites). We use this model to examine three pathogens with distinct environmental characteristics (influenza, rhinovirus, and norovirus) in four venue types. To parameterize the model for each pathogen we conducted a thorough literature search.

**Results:**

Based on parameter estimates from the literature the reproductive number ($\mathcal {R}_{0}$) for the fomite route for rhinovirus and norovirus is greater than 1 in nearly all venues considered, suggesting that this route can sustain transmission. For influenza, on the other hand, $\mathcal {R}_{0}$ for the fomite route is smaller suggesting many conditions in which the pathway may not sustain transmission. Additionally, the direct fomite route is more relevant than the hand-fomite route for influenza and rhinovirus, compared to norovirus. The relative importance of the hand-fomite vs. direct fomite route for norovirus is strongly dependent on the fraction of pathogens initially shed to hands. Sensitivity analysis stresses the need for accurate measurements of environmental inactivation rates, transfer efficiencies, and pathogen shedding.

**Conclusions:**

Fomite-mediated transmission is an important pathway for the three pathogens examined. The effectiveness of environmental interventions differs significantly both by pathogen and venue. While fomite-based interventions may be able to lower $\mathcal {R}_{0}$ for fomites below 1 and interrupt transmission, rhinovirus and norovirus are so infectious ($\mathcal {R}_{0}>>1$) that single environmental interventions are unlikely to interrupt fomite transmission for these pathogens.

**Electronic supplementary material:**

The online version of this article (10.1186/s12879-018-3425-x) contains supplementary material, which is available to authorized users.

## Background

While pathogens are sometimes transmitted by direct contact between infected and susceptible individuals, the environment is often an important mediator of transmission. Water, food, and fomites can act as environmental reservoirs, enhancing pathogens’ ability to be transmitted from host to host. A thorough understanding of these environmental pathways that affect risk can provide important opportunities for public health interventions.

Microbial risk assessment has a long history of focusing on risks associated with food and water transmission pathways. There is growing recognition, however, that many diseases previously considered to be primarily transmitted through direct contact, may in fact have significant fomite-mediated pathways. For example, norovirus, estimated to be responsible for 93% of nonbacterial gastroenteritis outbreaks in the United States [1], is transmitted not only by food and water, but also via contaminated surfaces. Influenza infection, annually costing greater than $10 billion in direct medical expenses [2], is transmitted through multiple routes: the air, direct droplet-spray (which may be thought of as direct contact), and potentially fomite-mediated transmission. Rhinovirus has also been shown to be transmitted through contaminated surfaces [3, 4]. Guidance on appropriate non-pharmaceutical interventions (e.g., masks, hand hygiene, and surface decontamination) depend on the dominant model of transmission [5].

In spite of the potential significance of fomite-mediated transmission, there is a longer tradition of environmental risk assessment and modeling environmental infection transmission through water [6, 7], food [8, 9], and even the air [10]; only recently has there been published work on fomite-mediated transmission [5, 11–14]. These fomite transmission models explicitly model pathogen transmission to susceptible hosts by assuming that pathogens in the environment only cause infection when they are eventually transferred to a susceptible host, an important departure from traditional transmission models that ignore pathogens in the environment. As such, this exposure pathway may be counteracted by: 1) natural background inactivation of the pathogen, 2) background clearance (e.g. air exchanges), and 3) intervention measures that aim to remove pathogen contamination (from air, water, hands, or surfaces). The effect of these measures may vary substantially between specific pathogens due to differences in environmental persistence and transfer efficiency from one medium to another.

To better understand fomite-mediated transmission across pathogens, we use microbiological and epidemiological data to inform a mechanistic compartmental model of the dynamics of contact and pathogen transfer between individuals via their hands and fomites, pathogen persistance in the environment, and pathogen shedding and recovery of infected individuals. We use this model to compare and contrast three viral pathogens (norovirus, influenza, and rhinovirus) that differ in their environmental persistence and shedding rates. We also examine the impact of venue contact rates, age groups present within the given setting, and contamination-accessible surfaces on transmission. We broadly conceptualized the differences in these variables as roughly corresponding to three general types of venues: subways, offices, and schools/daycares.

We selected three viral pathogens that varied in their persistence on hands and fomites to illustrate how different environmental sensitivities influence transmission dynamics. For each pathogen, we review the literature to summarize specific environmental transmission parameter values. Next, we use these pathogen-specific parameters to calculate the transmissibility of each pathogen in a range of venues. Finally, we ascertain the degree to which transmission of each pathogen may be controlled through environmental interventions (either hand hygiene or surface decontamination). Our focus here is a comparative analysis of fomite transmission across pathogens and venues. This analysis does not compare transmission that might occur by other routes, allowing us to focus on the impact of fomites as a reservoir of pathogens as well as interventions specifically targeting this pathway.

## Methods

### EITS model

Our model is an extension of the Environmental Infection Transmission System (EITS) modeling framework [12, 14]. Individuals are divided among susceptible (*S*), infectious (*I*) and removed (*R*) compartments. Pathogens that survive outside the host either contaminate a fomite (*F*) or individuals’ hands. We explicitly track the transfer of pathogens to and from hands and designate the hands compartments as the sum of all pathogens in susceptible hands (*H*_*S*_), infectious hands (*H*_*I*_) and removed hands (*H*_*R*_). We note that hand contamination is separate from infection status–a person who is infected may not have contaminated hands and a susceptible person may have contaminated hands. Individuals with contaminated hands may become infected through self-innoculation. The dynamics of the system are driven by the following events:

1. Inoculation. An individual’s hands, which could be contaminated with pathogens, touch the mouth and other membranes that could be a route of infection at a rate *ρ*. A fraction *χ* of the pathogens present in the hands (*H*_*S*_/*S*, in the case of susceptibles) are transferred to an exposure site (e.g., the nasal mucosa). The probability of infection is modeled by the linear dose-response function *P*. Therefore, new infections emerge at a rate *ρP*(*χH*_*S*_/*S*).
2. Fomite touching. Individuals touch fomites at a rate *ρ*_*T*_, exchanging pathogens on fomites and hands. Transfer efficiencies are denoted by *τ*_*FH*_ (fomite to hand) and *τ*_*HF*_ (hand to fomite). Due to the lack of bidirectional measurements, some mathematical models assume *τ*_*FH*_=*τ*_*HF*_. [14, 15]. However, recent studies have questioned the validity and consequences of this assumption [16]. Where bi-directional measurements were available, we parameterized these two transfer events separately. During a touching event, the amount of pathogens acquired is a function of the expected quantity of pathogen on *F*, the rate of effective touching (*ρ*_*T*_*τ*_*FH*_) and the size of the area touched by fingers (*κ*, proportional to *λ*), so that the overall transfer of pathogens from fomites to hands is *ρ*_*FH*_=*ρ*_*T*_*τ*_*FH*_*κ*, while *ρ*_*HF*_=*ρ*_*T*_*τ*_*HF*_.
3. Excretion. Shedding by an infectious individual (coughing, sneezing, exhaling, vomit, etc.) at a rate *α* contributes to contamination of surfaces and hands. *A* pathogen units are shed per excretion event, and a proportion *ϕ*_*H*_ of it is deposited in hands, while the remaining *ϕ*_*F*_=1−*ϕ*_*H*_ is collected in surfaces. Only a proportion *λ* of surfaces is accessible for contamination. The parameter for the proportion of accessible surfaces is an abstraction to represent that not all fomites can realistically be shed upon. The pathogen contamination rates to hands and surfaces are given by *α*_*H*_=*Aαϕ*_*H*_ and *α*_*F*_=*Aαϕ*_*F*_*λ*, respectively.
4. Pathogen inactivation (decay). Pathogens in the environment are inactivated at rate *μ*_*F*_ on fomites and *μ*_*H*_ on hands.
5. Recovery. Individuals transition from the *I* to the *R* class after recovery, at a rate *γ*.
6. Cleaning. Decontamination occurs at the hourly rate *Θ*_*F*_ on fomites and *Θ*_*H*_ on hands. Each cleaning event has an efficacy of *q*_*F*_ and *q*_*H*_. The product of these two terms (*θ*_*F*_ for fomites and *θ*_*H*_ for hands) gives the effective pathogen removal rate per hour due to the cleaning intervention.

We model these dynamics using the following ordinary differential equations (ODEs). The fomite compartment describes the contamination of fomites (averaged) in a particular venue and takes units of pathogen/hour. Table 1 provides a complete list of parameters.

1 $$ {{}\begin{aligned} \frac{dS}{dt} & = - \rho P(\chi H_{S}/S) S, \quad \quad \frac{dI}{dt} = \rho P(\chi H_{S}/S) S - \gamma I, \quad \quad \frac{dR}{dt} = \gamma I \\[.2cm] \frac{dF}{dt} & = a_{F} I - (\rho_{FH} N + \mu_{F} + \theta_{F}) F + \rho_{HF} (H_{S} + H_{I} + H_{R}) \\[.2cm] \frac{dH_{S}}{dt} & = \rho_{FH} S F - \mu_{H} H_{S} - \rho_{HF} S \tfrac{H_{S}}{S} - \theta_{H} H_{S} \\ &\quad - \rho S \left[ \tfrac{H_{S}}{S} P(\chi H_{S} / S) + \chi \tfrac{H_{S}}{S} (1 - P(\chi H_{S} / S))\right] \\[.2cm] & = \rho_{FH} S F - (\mu_{H} + \rho_{HF} + \chi \rho + \theta_{H}) H_{S} - (1-\chi) \rho P(\chi H_{S} / S) H_{S} \\[.2cm] \frac{dH_{I}}{dt} & = \rho_{FH} I F - \mu_{H} H_{I} - \rho_{HF} I \tfrac{H_{I}}{I} - \chi \rho I \tfrac{H_{I}}{I} - \theta_{H} H_{I}+ a_{H} I \\ &\quad+ (1-\chi) \rho H_{S} P(\chi H_{S} / S) - \gamma I \frac{H_{I}}{I} \\[.2cm] & = \rho_{FH} I F - (\mu_{H} + \rho_{HF} + \chi \rho + \theta_{H}) H_{I} + a_{H} I\\ &\quad+ (1-\chi) \rho P(\chi H_{S} / S) H_{S} - \gamma H_{I} \\[.2cm] \frac{dH_{R}}{dt} & = \rho_{FH} R F - \mu_{H} H_{R} - \rho_{HF} R \tfrac{H_{R}}{R} - \chi \rho R \tfrac{H_{R}}{R} - \theta_{H} H_{R} + \gamma H_{I} \\[.2cm] & = \rho_{FH} R F - (\mu_{H} + \rho_{HF} + \chi \rho + \theta_{H}) H_{R} + \gamma H_{I} \end{aligned}}  $$

**Table 1:** List of pathogen-specific parameters values and references used to produce Figs. 3 and 4

	Influenza		Rhinovirus		Norovirus	
**Pathogen-specific parameters**						
1/*γ*: Infectious	6	[38–40]	10.4	[41]	15	[42]
period (days)						
*α*: Shedding rate (pathogen hours ^−1^ people ^−1^)	1×10^4^	[43–45]	1×10^3^	[46, 47]	2.88×10^3^	[48, 49]
*μ*_*F*_: Inactivation rate	0.121	[50–52]	1.44	[53, 54]	0.288	[8]
in fomites (hours ^−1^)	(0.058, 0.121)		(0.990, 1.44)		(0.0006, 0.288)	
*μ*_*H*_: Inactivation rate	88.2	[13, 50, 52]	0.767	[55]	1.07	[9, 56, 57]
in hands (hours ^−1^)	(55.2, 88.2)				(0, 1.07)	
*τ*_*FH*_: Transfer	0.1	[13, 16, 50, 58]	0.2	[55, 59–61]	0.07	[62]
efficacy (*F* to *H*) (proportion)	(0.04, 0.16)		(0.1, 0.40)		(0.051, 0.089)	
*τ*_*HF*_: Transfer	0.025	[13, 16, 50, 58]	0.2	[55, 59–61]	0.13	[62]
efficacy (*H* to *F*) (proportion)	(0.01, 0.04)		(0.1, 0.40)		(0.094, 0.166)	
*ϕ*_*H*_: Pathogen	0.15		0.15		0.90	
excreted to H (proportion)	(0.10, 0.2)		(0.10, 0.2)		(0.50, 1)	
*ϕ*_*F*_: Pathogen	1−*ϕ*_*H*_		1−*ϕ*_*H*_		1−*ϕ*_*H*_	
excreted to F (proportion)						
*π*:Infectivity parameter in contact						
with x pathogens (unitless) ^*a*^	6.93e-05		2.46e-3		4.78e-4	
*a*_*H*_: rate pathogens are added to hands	*α* *ϕ*_*H*_		*α* *ϕ*_*H*_		*α* *ϕ*_*H*_	
(pathogen time ^−1^ people ^−1^)						
**Venue-specific parameters**						
*λ*: Accessible surfaces (proportion)	(0, 0.6)		(0, 0.6)		(0, 0.6)	
*κ*: fingertip to surface ratio per individual	$\frac {6e-06}{\lambda }$		$\frac {6e-06}{\lambda }$		$\frac {6e-06}{\lambda }$	
(1/people) ^*b*^						
*ρ*_*T*_: Rate of fomite touching (days ^−1^)	(0, 60)		(0, 60)		(0, 60)	
*ρ*_*FH*_: rate of pathogen pick up from fomites to hand	*ρ*_*T*_ *τ*_*FH*_ *κ*		*ρ*_*T*_ *τ*_*FH*_ *κ*		*ρ*_*T*_ *τ*_*FH*_ *κ*	
1/(days × people)						
*ρ*_*FH*_: rate of pathogen deposit from hand to fomite	*ρ*_*T*_ *τ*_*HF*_		*ρ*_*T*_ *τ*_*HF*_		*ρ*_*T*_ *τ*_*HF*_	
1/(days × people)						
*a*_*F*_: rate pathogens are added to fomites	*α* *ϕ*_*F*_ *λ*		*α* *ϕ*_*F*_ *λ*		*α* *ϕ*_*F*_ *λ*	
(pathogen days ^−1^ people ^−1^)						
**Cleaning parameters**						
*Θ*_*F*_: Rate of fomite cleaning (days ^−^1)	(0, 2)		(0, 2)		(0, 2)	
*Θ*_*H*_: Rate of hand cleaning (days ^−^1)	(0, 3)		(0, 3)		(0, 3)	
*q*_*F*_: Fomite cleaning efficacy (proportion)	1		1		1	
*q*_*H*_: Hand cleaning efficacy (proportion)	1		1		1	
*θ*_*F*_: Effective fomite cleaning rate	*q*_*F*_ *Θ*_*F*_		*q*_*F*_ *Θ*_*F*_		*q*_*F*_ *Θ*_*F*_	
*θ*_*H*_: Effective hand cleaning rate	*q*_*H*_ *Θ*_*H*_		*q*_*H*_ *Θ*_*H*_		*q*_*H*_ *Θ*_*H*_	
**Fixed parameters (across pathogens**						
**and venues)**						
*ρ*: Inoculation (hours ^−1^)	15.8	[23, 24]	15.8	[23, 24]	15.8	[23, 24]
*χ*: proportion of pathogens absorbed when	1		1		1	
self-inoculation occurs (proportion)						
^*a*^ Parameter fixed based on linearization of the dose-response curve						
^*b*^ Parameter fixed based on relative finger to body size.						

Point values appear on the left and references to the right. A range is also included for parameters that were used to perform a sensitivity analysis. Derived parameters are shown as a function of the parameters used to derive them. Bold headings are used to separate the table into subsections for legibility

**Fig. 1:**
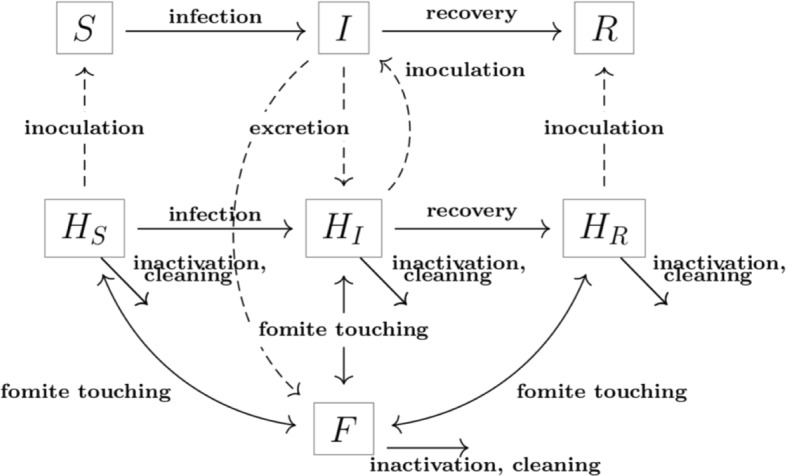
Model diagram Model tracks people (in compartments *S*, *I* or *R*) and pathogens on fomites (*F*) and hands (*H*_*S*_, *H*_*I*_, *H*_*R*_). The six events (inoculation, fomite touching, excretion, pathogen inactivation recovery and cleaning) are represented by arrows in the direction of the corresponding flow

Figure 1 summarizes the flows between compartments in the above equations. The dose response function *P*(*x*) gives the probability that a susceptible contracts the disease when inoculated with *x* units of pathogen from his/her hands. While a range of dose response functions have been proposed including linear (mass action), exponential, beta-Poisson, and Hill functions, these models yield similar results under low pathogen loads (small values of *x*). As a result, we use a linear dose response mechanism [17].

**Fig. 2:**
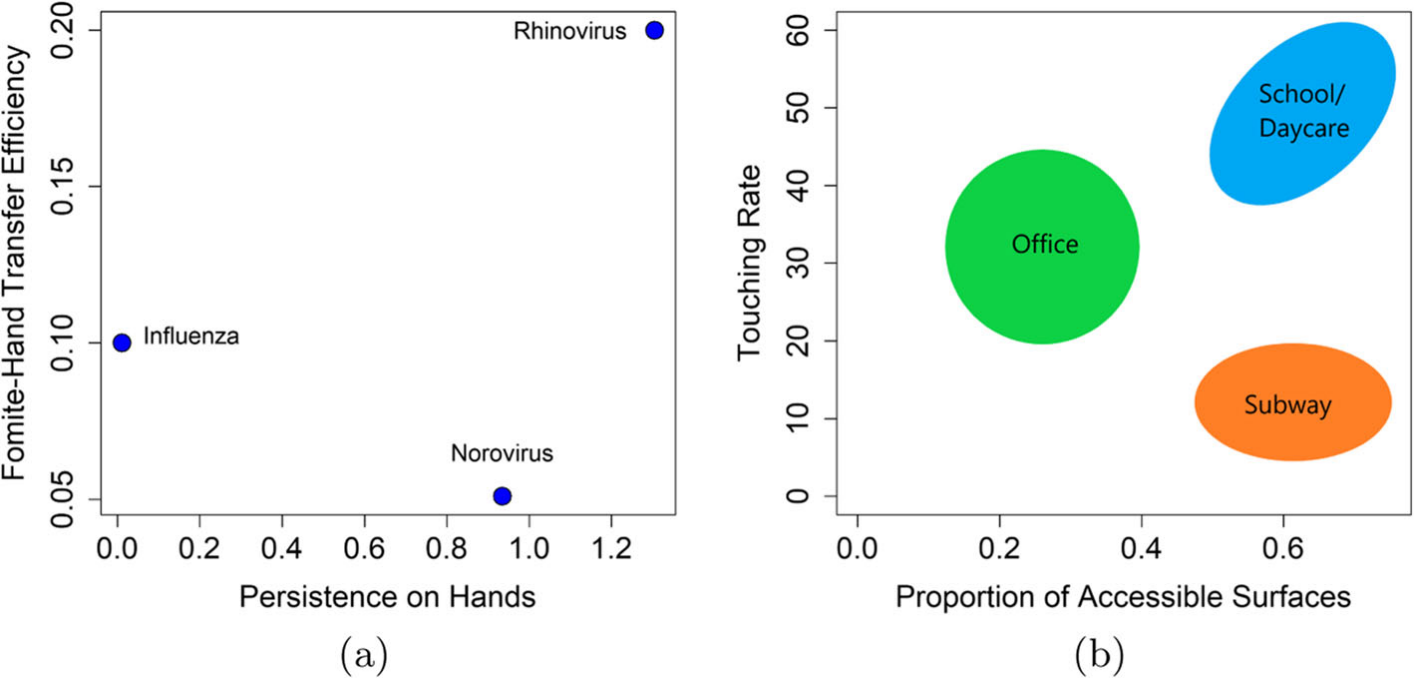
**a** The persistence duration on hands and fomite-hand transfer efficiency of the three pathogens examined. **b** Examples of three different venues as characterized by their proportion of accessible surfaces and the rate of contact with those surfaces

Our model parameters were based on both pathogen-specific characteristics (such as recovery rates, pathogen inactivation/persistence, pathogen excretion and pathogen dose response) and venue-specific characteristics (including surface touching rates and the amount of surfaces that could be contaminated with pathogen). A literature review was performed to obtain parameter values for influenza, rhinovirus, and norovirus (Table 1). We characterize pathogens based on their environmental persistence and transfer efficiencies in Fig. 2a.

Transmission venues are complex environments characterized both by their physical properties (e.g. types and quantity of fomites) and by the nature of host behaviors within these spaces (e.g. frequency of contact with fomites, the duration of time spent in a venue, or the density of hosts within the venue). Furthermore, risk within a venue may also vary by age group based on not only differences in contact rates but also shedding rates. For our analysis, we use a simplified representation of a venue, based three factors: the proportion of contamination-accessible fomites, shedding rates, and how frequently individuals interact with those fomites. For example, we characterize a daycare as a venue with a large fraction of accessible surfaces, high shedding rates, and a high contact rate between children and fomites. A subway is a compact environment that also has many surfaces that may be contaminated–but subway riders are in general older than daycare attendees and less likely to touch their environment as often due to perceived risk of disease. In contrast, an office setting in general will have fewer contaminated surfaces but likely higher touching rates. The parameter *λ* reflects the proportion of surfaces in a venue that are accessible to contamination, *ρ*_*T*_ controls the rate of fomite touching within a given venues, and *α* reflects the shedding rate. While we have no way of estimating the touching rates and proportion of accessible surfaces by venue directly, Fig. 2b summarizes where example venues are generally situated in our framework with respect to *λ* and *ρ*_*T*_. Because the parameter values corresponding to each type of venue are not known precisely, we simulate our model across the plausible range, using these values as general benchmarks to aid in interpreting our findings.

For this analysis, we treat each venue as a closed system and do not consider host movement. This approximation should not bias our results for venues in which the ratio of susceptible to infected individuals who come in and out of a venue remains constant. This assumption is likely to hold for settings like offices and daycares, for which the population that utilizes these venues is relatively constant over time, but might be more problematic for subways.

Finally, we include control measures parameters (cleaning rate and the proportion of pathogens killed by decontamination) to contrast the effectiveness of control measures among the pathogens and across venues. While the feasible frequency of cleaning is likely to vary by venue, we consider the same three frequencies for all three venues: 1 /2 days, daily, and twice daily. We expected that cleaning more often than twice per day was unlikely to be feasible for any of the three venues considered, and thus our interventions represent a theoretical upper bound on the effectiveness of surface decontamination interventions in these contexts. As with any mathematical model intended to capture a highly complex process, simplifying assumptions were included in our system. The population size is assumed to be constant (*N*=*S*+*I*+*R*), individuals are identical, except for the state they are in (Susceptible *S*, Infected *I* or Recovered *R*) and we do not include a latent (infectious) state. The *I* individuals are assumed to be the only source of infection, and new infections are produced only via self-inoculation; hand to hand spread of pathogens between individuals is not modeled. In addition, for this analysis we do not model the effect of imported cases in order to focus on the endogenous dynamics of our system. Finally, we assume that pathogens in fomites and fingertips distribute homogeneously, do not replicate and are instantaneously well mixed.

### Literature Review

We queried PubMed and Google Scholar to find sources for the biologically based parameters in our model: infectious period, inoculation rate, shedding rate, inactivation rate in fomites, inactivation rate in hands, transfer efficiency (fomite-hand), transfer efficacy (hand-fomite), and the proportion of pathogens excreted to hands.

### $\mathcal {R}_{0}$ and contribution of transmission routes

We calculate the reproductive number $\mathcal {R}_{0}$ for our model to characterize each pathogen’s outbreak potential across a range of venues as well as to evaluate the impact of environmental interventions. We use the next generation matrix method to compute $\mathcal {R}_{0}$ [18, 19]. We identified the transmission (new infections) and transition (changes in states, like removal, death or recovery) factors and linearized around the disease free equilibrium in order to define two matrices: (i) the matrix associated with transmission, which contains the rate at which infected individuals are produced; and (ii) the matrix associated with transitions, whose inverse contains the average lengths of time spent in each compartment. From these matrices, we identified $\mathcal {R}_{0}$ (the dominant eigenvalue of the product of the above defined matrices, see Additional file 1: Appendix for details).

The next generation approach yields an expression for $\mathcal {R}_{0}$ that agrees with the formula given in [14] and can be decomposed as follows [20–22].

2 $$ {{}\begin{aligned}\mathcal{R}_0 \,=\, P_{\text{{{inoculation}}}} P_{\text{{{pickup}}}} P'(0) \left[ \frac{a_{F}}{\gamma} + \frac{a_{H}}{\gamma} P_{\text{deposit}} \right] \,=\, \mathcal{R}_{0,F} + \mathcal{R}_{0,H},  \end{aligned}}  $$

where

$${{}\begin{aligned} \begin{array}{lll} &{}P_{\text{{{inoculation}}}} =& \frac{\chi \rho}{\mu_{H} + \rho_{HF} + \chi \rho + \theta_{H}}\\ &&\text{Proportion of pathogens on hands that are self} \\ && \text{ inoculated while still viable} \\[.2cm] &\!\!\!P_{\text{{{deposit}}}} =& \frac{\rho_{HF}}{\mu_{H} + \rho_{HF} + \rho \chi + \theta_{H}}\\ [4pt] &&\text{Proportion of pathogens on hands that are deposited} \\ && \text{ in fomites} \\[.2cm] &\!\!P_{\text{{{pickup}}}} =& \frac{\frac{\rho_{FH} N}{\rho_{FH} N + \mu_{F} + \theta_{F}}}{1 - \frac{\rho_{FH} N}{\left(\rho_{FH} N + \mu_{F} + \theta_{F}\right)} \frac{\rho_{HF}}{\left(\mu_{H} + \rho_{HF} + \chi \rho + \theta_{H}\right)}}\\ && \text{Pathogens on fomites that are picked up by hands} \\[.4cm] &\ \ \mathcal{R}_{0,F} =& \frac{a_{F}}{\gamma} P_{\text{{{inoculation}}}} P_{\text{{{pickup}}}} P'(0)\\ && \text{Direct fomite route contribution to} \mathcal{R}_0 \\[.3cm] &\ \mathcal{R}_{0,H} =& \frac{a_{H}}{\gamma} P_{\text{{{inoculation}}}} P_{\text{{{pickup}}}} P_{\text{{{deposit}}}} P'(0)\\ &&\text{Hand-fomite route contribution to} \mathcal{R}_{0}\\ \end{array} \end{aligned}} $$

Our model considers only fomite-mediated transmission, interactions between people such as handshake are ignored, meaning that pathogens must pass by a fomite to reach a susceptible. Using Eq. 2 we can analyze the two fomite mediated transmission routes (i) Direct fomite contamination route: ($\mathcal {R}_{0,F}: I \to F \to H_{S} \to S$) and (ii) Hand-fomite contamination route ($\mathcal {R}_{0,H}: I \to H_{I} \to F \to H_{S} \to S$). Essentially, the former component of $\mathcal {R}_{0}$ captures the contribution to infection from surfaces near an infected person, that become contaminated without the need for hand contact (for example, through droplet spray or vomiting directly on a surface), while the latter represents the contribution of contaminated hands of infected individuals when virus is then deposited to surfaces.

In our initial analysis we test the case where there is no intervention (i.e. *θ*_*H*_=*θ*_*F*_=0). We also set inactivation rates on fomites (*μ*_*F*_) and hands (*μ*_*H*_) to their maximum value observed in the literature to provide conservative $\mathcal {R}_{0}$ estimates. We note, however, that results were similar throughout the plausible range of inactivation for each pathogen for a common surface type (see Additional files).

Two types of interventions are considered in the model: (1) hand hygiene (*θ*_*H*_=*Θ*_*H*_*q*_*H*_) and (2) surface decontamination (*θ*_*F*_=*Θ*_*F*_*q*_*F*_). In both cases, the parameter associated to this measure is given by the product of frequency and efficacy of the cleaning event. In our simulations, we assume that all individuals (both infected and uninfected) wash their hands at the specified rate. Due to the deterministic nature of the model the control measures are applied continuously. The effectiveness is assessed by computing the control reproduction number $\mathcal {R}_{C}$, a reduced version of $\mathcal {R}_{0}$ due to the control measures that can be calculated using the equations presented above, where *θ*_*H*_ and *θ*_*F*_ are non-zero.

All code used to produce the results of this paper has been uploaded as additional materials.

## Results

### Literature Review

We relied on the literature to determine empirically plausible values for 8 model parameters (Table 1). Some of our parameter estimates required additional assumptions. While the parameters used in our model varied widely by pathogen, some of the ‘events’ we model also depended on other environmental factors, including humidity and surface porosity. We were not able to find detailed information about the influence of environmental factors on each pathogen separately. However, for all three pathogens, we had data for non-porous surfaces at relatively low humidity (20-40%). Therefore, we used parameters consistent with these environmental conditions in our simulations to allow us to make comparisons across pathogens and venues. Where a range of values for these environmental conditions were available, we used the maximum reported decay rate such that our estimates of the contribution of fomite transmission would be conservative. We describe our findings about the role of these other environmental factors on transmission parameters below. For the same reason, we also used the minimum shedding rate reported in the experimental literature.

Our model considered two behavioral parameters: the rate of self-innoculation (*ρ*, face-touching events) and the rate of fomite touching (*ρ*_*T*_). We used the same two sources for the self-innoculation rate for all three pathogens [23, 24] as this rate does not appear to change meaningfully between pathogens. We assumed that the rate of fomite touching is more strongly determined by venue. We simulated across a range of fomite touching rates, with the lowest touching rates expected in subways and much higher touching rates in daycare settings (Fig. 2b).

Another parameter for which we found no information in the literature review was the fraction of pathogens shed to hands (*ϕ*_*H*_) versus directly into the environment for our pathogens of interest. We assumed that this fraction would vary depending on the mechanism of shedding (i.e. coughing openly vs. vomiting). We argue that gastrointestinal pathogens would have a higher fraction of shedding to hands vs. fomites due to the more localized nature of GI shedding events. Thus, our model is parameterized such that norovirus shedding contaminates hands more than rhinovirus or influenza.

For the remaining 6 parameters in our model (*γ*,*μ*_*F*_,*μ*_*H*_,*τ*_*FH*_,*τ*_*HF*_, and *α*), we identified 10, 7, and 6 articles for influenza, rhinovirus, and norovirus respectively. When meta-analyses were available, these estimates were used instead of individual parameter estimates. Thus the number of unique empirical studies used to inform our model is somewhat higher. References corresponding to each of these parameter values are shown in Table 1.

**Infectious period (1/*γ*)** The infectious period of each pathogen has been relatively well-studied. Notably, all three pathogens exhibit shedding beyond their symptomatic period, with norovirus having the longest total duration of shedding.

**Inactivation rates on fomites and hands (*μ***_***F***_** and*μ***_***H***_**)** For all pathogens, the inactivation rates on fomites were highly variable by surface, with higher inactivation rates on hands (which are a porous surface). Influenza, the only pathogen for which decay rates were available for porous environmental surfaces besides stainless steel, had much higher inactivation rates on porous surfaces. Notably, some pathogens exhibited biphasic inactivation, with faster initial inactivation followed by a period of slow inactivation or persistence without measurable decay. When this occurred, we used the average inactivation estimates over the first hour, when decay rates were highest, to parameterize our model. Influenza appears to survive for the shortest amount of time on hands, with an order of magnitude higher inactivation rate than either rhinovirus or norovirus. While inactivation rates on fomites were relatively insensitive to temperature, they were more sensitive to changes in humidity, with drier conditions generally promoting higher inactivation rates. The exception was influenza, which appeared to survive better at low humidity.

**Transfer efficiencies (*τ***_***F*,*H***_**,*τ***_***H*,*F***_**)** For influenza and norovirus, transfer efficacies appear to be asymmetrical. Influenza transfers more readily from fomites to hands than hands to fomites. The reverse appears to be true for norovirus. However, studies of rhinovirus do not appear to measure directional transfer. For influenza, transfer efficiency was also lower for porous than non-porous surfaces.

**Shedding rate (*α*)** Shedding concentrations varied considerably between pathogens as well as between individuals for a given pathogen. We modeled the overall shedding rate as the product of the concentration of pathogen shed per event, the volume of fluid excreted per event, and the number of shedding events. An example of this calculation can be found in the Additional file 1: Appendix. The average shedding rate for influenza was found to be an order of magnitude higher than for rhinovirus and norovirus. Existing studies characterizing the volume and frequency of shedding events were available for influenza and norovirus, but not rhinovirus. Given that rhinovirus causes similar upper respiratory tract symptoms as influenza, we used the same parameterization for these two pathogens for volume of fluid excretion and number of shedding events, but influenza had a higher concentration per shedding event. While viral shedding rates were similar for all age groups for both rhinovirus and norovirus, very young children (< 1 year) had higher influenza shedding rates [25–27]. Between-individual shedding rates were high among young children and we were only able to find estimates from one small study, which suggested that young children might shed up 2 orders of magnitude more than older individuals [25]. Both because data describing differences in shedding rates were sparse and because this difference in shedding was only found for young children who are less mobile and may interact with their environment in different ways, we used the same shedding rates for all age groups and venues in our simulations. However, qualitatively, this increased shedding in children would tend to increase risk of fomite-mediated transmission in daycare settings.

### Outbreak potential ($\mathcal {R}_{0}$) by pathogen and venue

**Fig. 3:**
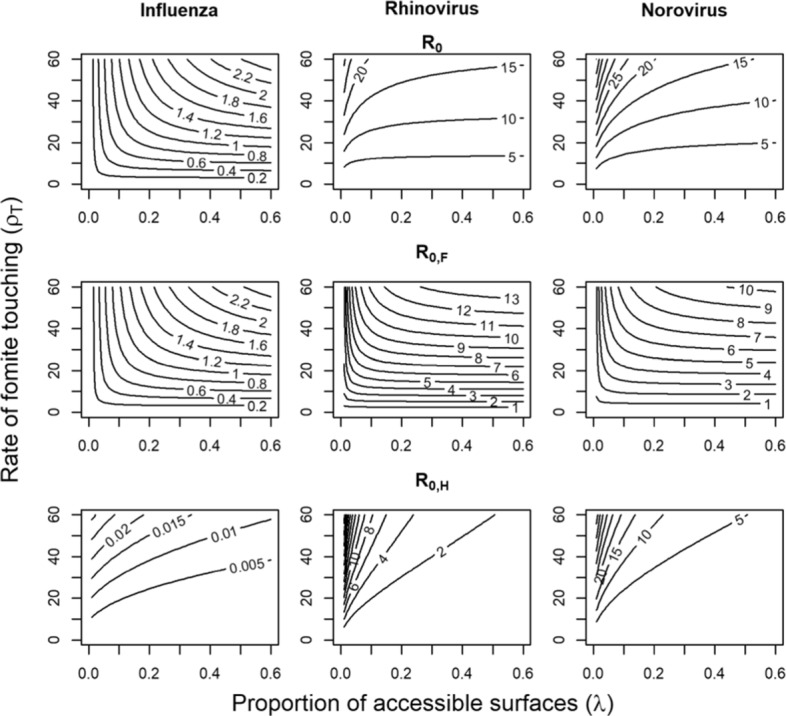
Reproduction numbers $\mathcal {R}_{0}$ as a function of fomite touching rate (*ρ*_*T*_) and proportion of touchable surfaces (*λ*)

Behavior and venue are important drivers of transmission. For influenza, transmission via the fomite route is only sustainable for venues with high touching rates (*ρ*_*T*_>20) such as child care centers (Fig. 3, column 1). Airborne transmission may therefore be more likely to sustain influenza transmission in venues where either the touching rate is low (e.g. offices) or proportion of accessible surfaces is very low (e.g. outdoor venues). By contrast, our model suggests that rhinovirus and norovirus transmission by the fomite pathway are sustainable in nearly all venues (Fig. 3, columns 2 and 3). In all venues characterized by our chosen range of touching rates and accessible surface fractions, norovirus has the highest overall $\mathcal {R}_{0}$, followed by rhinovirus with influenza having the lowest overall $\mathcal {R}_{0}$. While norovirus and rhinovirus shed fewer viral copies than influenza, they have much longer infectious periods, as well as longer persistence on hands.

When we examine the two components of $\mathcal {R}_{0}$ ($\mathcal {R}_{0,F}$ and $\mathcal {R}_{0,H}$), we see that the direct fomite route is most important for transmission of influenza (Fig. 3, row 2), whereas the hand-fomite route was more important for rhinovirus and norovirus (see Fig. 3). Based on our sensitivity analyses (not shown), for norovirus and influenza, the relative importance of each pathway was highly sensitive to the fraction of pathogens shed onto hands rather than surfaces. When a larger proportion of pathogens was shed onto surfaces, the direct fomite route became more important. For rhinovirus, the fraction of pathogens shed to hands (*ϕ*_*H*_) was less uncertain and altering this quantity did not greatly impact the relative importance of the two pathways. The reason the hand-fomite route dominated for rhinovirus is due to its relatively larger transfer efficiency proportion and low inactivation rate on hands.

The overall reproduction number for both pathways combined is strongly impacted by the shedding rate, *α*. In particular, the shedding rate acts as an effect modifier on $\mathcal {R}_{0}$. When this shedding rate is high, the overall reproduction number can remain high, even when persistence on hands and fomites is low. For norovirus in particular, small decreases in the overall shedding rate led to large decreases in the overall reproduction number of this pathogen. In contrast, although influenza had the highest shedding rate, our model suggests it had the lowest transmissibility through the fomite route, largely because of its rapid inactivation on hands and low transfer efficiencies (*τ*_*HF*_ and *τ*_*FH*_).

### Risk reduction by cleaning strategies

**Fig. 4:**
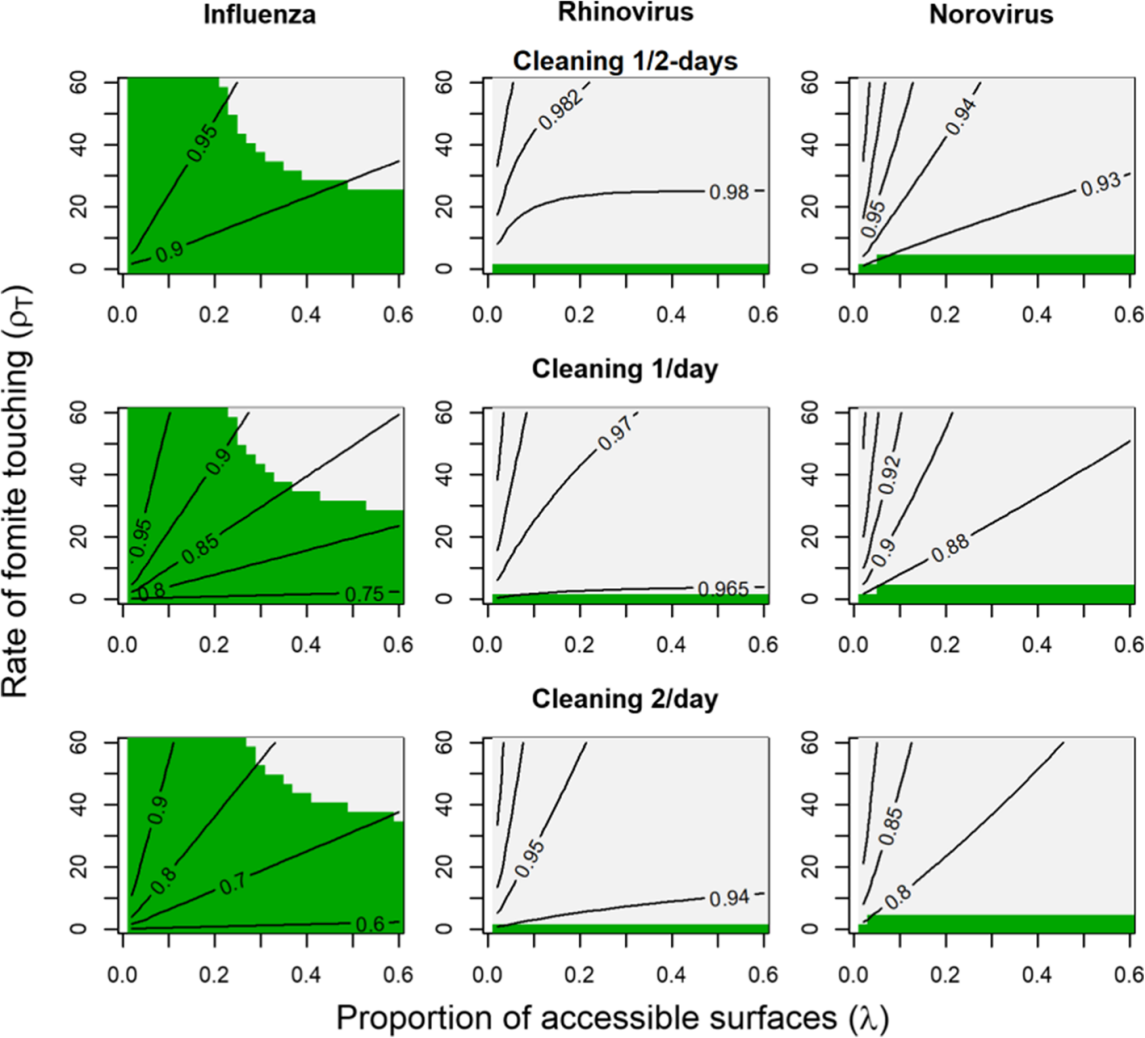
Contours for the ratio $\frac {R_{C}}{R_{0}}$ for cleaning strategies consisting of hand washing three times a day and surface decontamination at varying frequency (Solid lines). Green regions indicate venues where *R*_*C*_<1, i.e. the interventions successfully prevent an outbreak. The results for each pathogen (influenza, rhinovirus, and norovirus) are summarized by column, while different strategies are shown by row. Surface cleaning is performed every two days (top row), once a day (middle row), or two times a day (bottom row). Hand washing occurs three times per day for each row

Our results indicate that higher reductions on $\mathcal {R}_{0}$ can be achieved by increasing the frequency of surface decontamination (Fig. 4). In contrast, hand washing did not have an appreciable effect on $\mathcal {R}_{0}$. In Fig. 4, we show effects for three times daily hand washing only. As a sensitivity analysis, we also considered increasing the frequency of hand washing to hourly and found very similar results.

For influenza, only higher frequency (≥ 1/day) surface decontamination strategies appear to meaningfully reducing $\mathcal {R}_{0}$, with a maximum reduction of 40% in low surface contact venues. However, from Fig. 3, fomite transmission is only possible in settings with higher touching rates and proportions of accessible surfaces. Thus, surface decontamination for influenza may prevent outbreaks in venues with moderate surface contact rates and many accessible surfaces. In contrast, in our simulations similar interventions for rhinovirus and norovirus were not effective, even with cleaning frequencies of up to twice per day. For rhinovirus, an effect of no more than 5% is observed and for norovirus the maximum effect size was 20%. Even with this 20% effect size, our model suggests that $\mathcal {R}_{0}$ would remain substantially above 1. While higher cleaning frequencies may demonstrate improved efficacy, they are likely not practical to implement and so were not considered in this analysis.

## Discussion

Fomites are an important source reservoir for pathogens that can persist in the environment. Environmentally persistent pathogens like norovirus and rhinovirus are able to exploit fomite pathways in a variety of potential venues. For pathogens with higher surface die-off rates such as influenza, fomite transmission is sustainable in a narrower range of venues. Based on these findings, the fomite-mediated pathway may be sufficient to sustain transmission even if interventions targeting more direct pathways are successful.

Focusing on fomite-mediated routes allow us to examine a transmission pathway that might otherwise be masked by faster processes such as direct transmission and provides a natural way to evaluate the effectiveness of environmental interventions such as surface decontamination. While we do not consider transmission that might occur through other routes, we show that the extent to which environmental interventions can successfully control fomite-mediated transmission is affected by both the venue in which transmission occurs (both physical properties and behavioral factors) and intrinsic pathogen properties (including inactivation rates, transfer efficiencies, and shedding rate) (Additional file 2).

### Characterizing pathogens

The impact of interventions on fomite-mediated transmission varied by pathogen. For influenza, high-frequency surface decontamination could prevent outbreaks by reducing $\mathcal {R}_{0}$ for the fomite route below 1 in venues with high proportions of accessible surfaces and moderate surface contact rates. This is because influenza demonstrated the lowest overall fomite $\mathcal {R}_{0}$ in our analysis. However, even in venues that are not favorable for fomite transmission, influenza may still be able to maintain transmission by inhalation of aerosolized viral particles or direct droplet spray, necessitating additional control strategies. Notably, it may be important to tailor environmental interventions to specific venues, as the effect of a given influenza transmission mechanism may not be consistent between venues with different environmental properties [5]. For rhinovirus and norovirus, none of the interventions considered had an appreciable effect, suggesting that alternative strategies may be needed to control these pathogens. These results are consistent with recent work by Lei et al, who found that the fomite-mediated route was important for norovirus, but less so for influenza [28]. To be effective, surface decontamination interventions for norovirus and rhinovirus may need to be more frequent (> 1x /day), tailored to the specific context, and timed early in outbreaks to interrupt transmission. These differences were driven by the interaction between multiple properties of the pathogens. Because of its low transfer efficiency, high inactivation rates on hands, and relatively short duration of infectiousness, influenza had the lowest $\mathcal {R}_{0}$ for the fomite route, making fomite-mediated transmission easier to control despite its high shedding rate. Both rhinovirus and norovirus were more efficiently transferred, had high persistence on both hands and fomites, and produce longer periods of shedding, making the transmission potential high for both pathways and consequently more difficult to control, even with frequent surface decontamination.

Our model parameterization is derived from pathogen data from existing literature. While this allows us to examine concrete scenarios, the underlying data is subject to substantial uncertainty. Resolving this uncertainty will require additional empirical studies, particularly for factors affecting pathogen inactivation rates (e.g., humidity, surface porousity, etc.) and transfer efficiencies. Many of the commonalities shown here in our literature review may not hold for other pathogens and more work is needed to determine which pathogen features contribute to their different behavior on surfaces. For example, while our literature review found that low humidity promoted higher inactivation for rhinovirus and norovirus, for influenza the association is in fact reversed, with higher humidity resulting in high inactivation rates. In addition, while transmission potential from nonporous surfaces is generally lower for many bacteria due to increased inactivation rates on and lower transfer efficiencies from those surfaces, we only had data from multiple surface types for influenza [29, 30]. Given that prior work has also shown that certain viruses, like polio, do not exhibit these same trends, we did not assume that these same trends held for rhinovirus and norovirus and more experiments are needed to verify these patterns [29].

### Defining venues

Transmission dynamics are strongly dependent on the context in which that transmission occurs. In our model, the important factors governing these contexts are their physical properties, e.g. the degree to which surfaces can be contaminated by shedding events and behavioral properties e.g., how frequently those fomites are touched. It is important to note that interpreting a transmission venue in our framework depends on the method of shedding. For example, norovirus is primarily shed through contaminated fecal matter and vomiting. These shedding events may occur at a lower rate than coughing episodes that can spread respiratory pathogens and are also more likely to be confined to smaller locations, such as bathrooms. In reality a given location may be comprised of multiple sub-venues with differing transmission potentials, e.g. an office floor has both high potential locations for norovirus such as restrooms, and lower potential locations such as open work spaces.

These dimensions of transmission venues can be addressed by different interventions. For example, individual based interventions might focus on reducing fomite touching whereas centralized interventions change the surfaces available for contamination. In general, we found that $\mathcal {R}_{0}$ for the fomite route was more sensitive to surface properties than to the rate of touching fomites. By extension, interventions that improve surface decontamination may be more effective than hand washing interventions when fomites are the primary transmission route. Specifically, when the proportion of accessible surfaces (*λ*) was low (such as in an office or subway scenario), touching rates were most important for determining fomite $\mathcal {R}_{0}$ because surfaces were highly concentrated. However, at higher values of *λ*, touching rates did not contribute as much to fomite $\mathcal {R}_{0}$. Of the four venues considered, the highest values of $\mathcal {R}_{0}$ for the fomite route occurred in settings like schools and daycares, where both the proportion of accessible surfaces and the touching rates were high leading to frequent exposure to surfaces with somewhat lower concentration of pathogen. Daycares may be an even greater source of risk for pathogens that have higher shedding rates among children. These venues are likely to be of particular interest for infection control efforts as non-pharmaceutical intervention and environmental microbiology studies have indicated that pathogen persistence on school and day care surfaces could be an important source of infections [31, 32]. Additionally, healthcare settings exhibit many features of high contact and high accessible surface venues. Indeed, fomite-targeted interventions are important as preventative measures to reduce nosocomial infections [33–35].

### Future work

Given our specific interest in fomite-mediated transmission, we have chosen to ignore transmission that might occur through other routes, including direct hand to hand contact. Leaving out this pathway may contribute to our finding that hand washing appears to be less efficacious at reducing transmission than surface decontamination. Future studies could extend our work by including direct transmission through hand to hand contact. Our fomite-mediated transmission framework could be extended to address further aspects of host behaviors in venues. In particular, venues vary considerably in terms of the density of hosts present both between types of venues, and over time within a given venue. Additionally, hosts move between multiple venues in a given day, limiting the total amount of time in each venue. Finally, host behaviors may change upon infection (e.g. staying home due to symptoms), which may impact their exposure to potential co-infections or sequential reinfection. Addressing these factors will likely require a stochastic, individual based model, as variation in host behavior can make ODE models computationally inefficient and difficult to analyze.

Whenever possible, we have chosen to make assumptions and use parameter values that would tend to minimize the potential contribution of the fomite route, so that our results would be a lower bound. For example, we used the lowest transfer rates and highest shedding rates available for nonporous surfaces, we assumed that all individuals washed their hands with the handwashing intervention (both infected and uninfected), and we did not consider heterogeneity of fomite touching rates within a given venue. All of these assumptions would tend to decrease $\mathcal {R}_{0}$. For heterogeneity in touching rates within a venue, we know from other work that when heterogeneity is high (for example, the presence of key fomites with high touching rates in a venue with generally low touching rates), its presence will generally increase the variance of $\mathcal {R}_{0}$ within a given setting. In a deterministic framework, this pattern tends to lead to a higher average $\mathcal {R}_{0}$ whereas in a stochastic framework it leads to more stochastic die out of outbreaks, but also increased outbreak size when outbreaks do occur [36].

The main exception to this generally conservative approach is that we relied on data for non-porous surfaces to parameterize our model, which generally have lower inactivation rates and higher transfer rates than porous surfaces. The difference in transmission between porous and non-porous surfaces is likely to have a larger impact on pathogens that are already close to the threshold for fomite transmission, such as influenza, and is probably less crucial for other pathogens, like norovirus and rhinovirus, where $\mathcal {R}_{0}$ for the fomite route is far greater than 1. In office and subway settings, a large proportion of environmental fomites are non-porous, whereas many fomites are porous in daycare settings. Future studies should explore the extent to which pathogen persistence and transfer efficiency varies by surface. These data would would allow for a more sophisticated analysis of the potential for fomite transmission across different venues.

## Conclusions

Fomite-mediated transmission introduces both challenges and opportunities for infection control due to interactions between the properties of pathogens and venues. Our analysis has shown that fomites can be an important source of risk for pathogens that are often considered to be, primarily, directly transmitted. In particular, we found that fomite-mediated transmission is dependent on both behavioral factors influencing contact with fomites as well as the physical environment and surfaces available for contamination in each venue. This result underscores the need to think critically about how a venue is characterized from a transmission perspective in order to design interventions that can appropriately target key stages in the transmission process. Overall, our analysis provides a useful conceptual framework for considering fomite-mediated transmission as a pathway that can be used in other environmental models. Our use of empirical studies to parameterize the transmission model offers an important link between laboratory and modeling studies. Empirical studies offer substantial information for use in parameterizing mechanistic models, while constructing these models can also reveal which biological parameters have not been well studied, or show substantial variation between studies.

When appropriately constrained by pathogen- and venue-specific data, transmission models that explicitly account for fomite-mediated transmission can be a useful tool to compare transmission potential across different pathogens and different venues.

## Additional files

Additional file 1Appendix. The appendix shows the derivation of the $\mathcal {R}_{0}$ calculations. (PDF 607 kb)

Additional file 2 Code Files. This file contains the code used for generating figures 1, 3, and 4. (DOCX 22 kb)
