# Supplementary material for: Fomite-mediated transmission as a sufficient pathway: a comparative analysis across three viral pathogens
Source: BMC Infect Dis. 2018 Oct 29;18:540. doi: 10.1186/s12879-018-3425-x (PMC6206643; doi:10.1186/s12879-018-3425-x)
Supplement: Supplementary file 1 — Appendix. The appendix shows the derivation of the $\mathcal {R}_{0}$R0 calculations. (PDF 607 kb) [file 12879_2018_3425_MOESM1_ESM.pdf]

Appendix

immediate

October 5, 2017

Model Equations and Parameters

The ODEs governing the dynamics of our model are given by

$$\begin{aligned}
\frac{dS}{dt} &= -\rho P(\chi H_S/S)S, \qquad \frac{dI}{dt} = \rho P(\chi H_S/S)S - \gamma I, \qquad \frac{dR}{dt} = \gamma I \\
\frac{dF}{dt} &= a_F I - (\rho_{FH}N + \mu_F + \theta_F)F + \rho_{HF}(H_S + H_I + H_R) \\
\frac{dH_S}{dt} &= \rho_{FH}SF - \mu_H H_S - \rho_{HF}S\frac{H_S}{S} - \theta_H H_S - \rho S\left[\frac{H_S}{S}P(\chi H_S/S) + \chi\frac{H_S}{S}(1 - P(\chi H_S/S))\right] \\
&= \rho_{FH}SF - (\mu_H + \rho_{HF} + \chi\rho + \theta_H)H_S - (1-\chi)\rho P(\chi H_S/S)H_S \\
\frac{dH_I}{dt} &= \rho_{FH}IF - \mu_H H_I - \rho_{HF}I\frac{H_I}{I} - \chi\rho I\frac{H_I}{I} - \theta_H H_I + a_H I + (1-\chi)\rho H_S P(\chi H_S/S) - \gamma I\frac{H_I}{I} \\
&= \rho_{FH}IF - (\mu_H + \rho_{HF} + \chi\rho + \theta_H)H_I + a_H I + (1-\chi)\rho P(\chi H_S/S)H_S - \gamma H_I \\
\frac{dH_R}{dt} &= \rho_{FH}RF - \mu_H H_R - \rho_{HF}R\frac{H_R}{R} - \chi\rho R\frac{H_R}{R} - \theta_H H_R + \gamma H_I \\
&= \rho_{FH}RF - (\mu_H + \rho_{HF} + \chi\rho + \theta_H)H_R + \gamma H_I
\end{aligned} \tag{1}$$

{main}

Additional parameters used in the model are shown in the table below.

1 Derivation of $\mathcal{R}_0$

The compartments of model (1) are classified in infectious (I, F, H_S, H_I, H_R) and non infectious (S, R). Then, the matrix F and T associated to new infections and transitions are computed

$$F = \begin{bmatrix} 0 & 0 & \chi\rho P'(0) & 0 & 0 \\ 0 & 0 & 0 & 0 & 0 \\ 0 & 0 & 0 & 0 & 0 \\ 0 & 0 & 0 & 0 & 0 \\ 0 & 0 & 0 & 0 & 0 \end{bmatrix},$$

$$V = \begin{bmatrix} \gamma & 0 & 0 & 0 & 0 \\ -a_F & \rho_{FH}N + \mu_F + \theta_F & -\rho_{HF} & -\rho_{HF} & -\rho_{HF} \\ 0 & -\rho_{FH}N & \mu_H + \rho_{HF} + \chi\rho + \theta_H & 0 & 0 \\ -a_H & 0 & 0 & \mu_H + \rho_{HF} + \chi\rho + \theta_H + \gamma & 0 \\ 0 & 0 & 0 & -\gamma & \mu_H + \rho_{HF} + \chi\rho + \theta_H \end{bmatrix}$$

Table 1: List of parameters and units

Parameter	Description	Units
ρ	rate of self inoculation	1/time
χ	proportion of pathogens absorbed when self inoculation occurs	unitless
γ	recovery rate	1/time
μ_F, μ_H	pathogen decay rate in fomite, hands	1/time
ρ_T	rate of fomite touching	1/time
τ_{FH}, τ_{HF}	transfer efficacy	unitless
κ	fingertip to surface ratio per individual	1/people
ρ_{FH}	($\rho_{FH} = \rho_T \tau_{FH} \kappa$) rate of pathogen pick up from fomites to hand	1/(time * people)
ρ_{HF}	($\rho_{HF} = \rho_T \tau_{HF}$) rate of pathogen deposit from hands to fomite	1/time
A	amount of pathogens released per excretion	pathogen/people
α	rate of pathogens excretion	1/time
λ	proportion of touchable surfaces	unitless
ϕ_F, ϕ_H	proportion of pathogen excreted to fomite, hands	unitless
a_F	($a_F = A\alpha\phi_F\lambda$) rate pathogens are added to fomite	pathogen/(time * people)
a_H	($a_H = A\alpha\phi_H$) rate pathogens are added to hands	pathogen/(time * people)
Θ_H	Cleaning rate (hands)	1/time
q_H	Cleaning efficacy in hands	unitless
θ_H	$= q_H\Theta_H$Cleaning exit rate in hands	1/time
Θ_F	Cleaning rate (fomite)	1/time
q_F	Cleaning efficacy in fomite	unitless
θ_F	$= q_H\Theta_H$ Cleaning exit rate in fomite	1/time
$pi = P'(0)$	Infectivity parameter in contact with x pathogens	unitless

{Table_par[illegible]

The $\mathcal{R}_0$ is the dominant eigenvalue of the matrix

$$FV^{-1} = \begin{bmatrix} \chi\rho P'(0)V_{31}^{-1} & * & * & * & * \\ 0 & 0 & 0 & 0 & 0 \\ 0 & 0 & 0 & 0 & 0 \\ 0 & 0 & 0 & 0 & 0 \\ 0 & 0 & 0 & 0 & 0 \end{bmatrix},$$

where V_{31}^{-1} is the (3,1) entry of the inverse matrix of V, and is given by

$$\begin{aligned} V_{31}^{-1} &= \frac{\rho_{FH}N[a_H\rho_{HF}\gamma + (\mu_H + \rho_{HF} + \chi\rho + \theta_H)(a_F(\mu_H + \rho_{HF} + \chi\rho + \theta_H + \gamma) + \rho_{HF}a_H)]}{\gamma(\mu_H + \rho_{HF} + \chi\rho + \theta_H + \gamma)(\mu_H + \rho_{HF} + \chi\rho + \theta_H)[(\rho_{FH}N + \mu_F + \theta_F)(\mu_H + \rho_{HF} + \chi\rho + \theta_H) - \rho_{HF}\rho_{FH}\text{[illegible]}} \\ &= \cdots = \frac{1}{\gamma(\mu_H + \rho_{HF} + \chi\rho + \theta_H)}\left[a_F P_{\text{pickup}} + a_H P_{\text{pickup}} P_{\text{deposit}}\right]. \end{aligned}$$

The dominant eigenvalue of this matrix is the only non zero element in the diagonal, for this reason other non relevant entries of the matrix have been denoted by $*$. Thus,

$$\mathcal{R}_0 = \chi\rho P'(0)V_{31}^{-1} = P_{\text{inoculation}} P_{\text{pickup}}\left[\frac{a_F}{\gamma} + \frac{a_H}{\gamma}P_{\text{deposit}}\right] = \mathcal{R}_{0,F} + \mathcal{R}_{0,H},$$

where

$$P_{\text{deposit}} = \tfrac{\rho_{HF}}{(\mu_H + \rho_{HF} + \rho\chi + \theta_H)}, \quad P_{\text{inoculation}} = \tfrac{\chi\rho P'(0)}{\mu_H + \rho_{HF} + \chi\rho + \theta_H}, \quad P_{\text{pickup}} = \frac{\frac{\rho_{FH}N}{(\rho_{FH}N + \mu_F + \theta_F)}}{1 - \frac{\rho_{HF}}{(\mu_H + \rho_{HF} + \chi\rho + \theta_H)}\frac{\rho_{FH}N}{(\rho_{FH}N + \mu_F + \theta_F)}}$$

2 Simplification to SIR

If we assume mass action incidence $P(x) = \pi x$ then the system becomes

$$\begin{aligned} \frac{dS}{dt} &= -\rho\pi\chi\frac{H}{N}S, \qquad \frac{dI}{dt} = \rho\pi\chi\frac{H}{N}S - \gamma I, \qquad \frac{dR}{dt} = \gamma I \\ \frac{dF}{dt} &= a_F I - (\rho_{FH}N + \mu_F)F + \rho_{HF}H \\ \frac{dH}{dt} &= \rho_{FH}NF - (\mu_H + \rho_{HF} + \chi\rho)H + a_H I \end{aligned}$$

Assuming that pathogens reach equilibrium before the population does, we obtain

$$\frac{dH}{dt} = 0 \qquad \rightsquigarrow \qquad H = \frac{\rho_{FH}N}{\mu_H + \rho_{HF} + \chi\rho}F + \frac{a_H}{\mu_H + \rho_{HF} + \chi\rho}I,$$

and

$$\begin{aligned} \frac{dF}{dt} &= a_F I - (\rho_{FH}N + \mu_F)F + \rho_{HF}\left(\tfrac{\rho_{FH}N}{\mu_H+\rho_{HF}+\chi\rho}F + \tfrac{a_H}{\mu_H+\rho_{HF}+\chi\rho}I\right) \\ &= \left(a_F + \tfrac{a_H\rho_{HF}}{\mu_H+\rho_{HF}+\chi\rho}\right)I - \left[\rho_{FH}N\left(1 - \tfrac{\rho_{HF}}{\mu_H+\rho_{HF}+\chi\rho}\right) + \mu_F\right]F \\ &= \left(a_F + \tfrac{a_H\rho_{HF}}{\mu_H+\rho_{HF}+\chi\rho}\right)I - \left[\rho_{FH}N\left(\tfrac{\mu_H+\chi\rho}{\mu_H+\rho_{HF}+\chi\rho}\right) + \mu_F\right]F \end{aligned}$$

so that

$$\frac{dF}{dt} = 0 \qquad \rightsquigarrow \qquad F = \frac{a_F + \frac{a_H\rho_{HF}}{\mu_H+\rho_{HF}+\chi\rho}}{\rho_{FH}N\left(\frac{\mu_H+\chi\rho}{\mu_H+\rho_{HF}+\chi\rho}\right) + \mu_F}\, I$$

and

$$\begin{aligned} H &= \frac{1}{\mu_H + \rho_{HF} + \chi\rho}(\rho_{FH}NF + a_H I) = \frac{1}{\mu_H + \rho_{HF} + \chi\rho}\left[\rho_{FH}N\frac{a_F + \frac{a_H\rho_{HF}}{\mu_H+\rho_{HF}+\chi\rho}}{\rho_{FH}N\left(\frac{\mu_H+\chi\rho}{\mu_H+\rho_{HF}+\chi\rho}\right) + \mu_F} + a_H\right]I \\ &\vdots \\ &= \frac{1}{\mu_H + \rho_{HF} + \chi\rho}\left[P_{\text{pickup}}\left(a_F + a_H P_{\text{deposit}}\right) + a_H\right]I, \end{aligned}$$

Finally, replace the above formula in the the derivative of S to obtain an expression in terms of S and I

$$\begin{aligned} \frac{dS}{dt} &= -\rho\pi\chi\frac{H}{N}S = -\frac{\rho\pi\chi}{N}\left(\frac{1}{\mu_H + \rho_{HF} + \chi\rho}[P_{\text{pickup}}\left(a_F + a_H P_{\text{deposit}}\right) + a_H]I\right)S \\ &= \frac{P_{\text{inoculation}}}{N}[P_{\text{pickup}}\left(a_F + a_H P_{\text{deposit}}\right) + a_H]SI \end{aligned}$$

to obtain

$$\mathcal{R}_0 = \frac{1}{\gamma}P_{\text{inoculation}}[P_{\text{pickup}}\left(a_F + a_H P_{\text{deposit}}\right) + a_H] = \mathcal{R}_F + \mathcal{R}_{HF} + \mathcal{R}_H.$$

3 Sensitivity Analysis

For our sensitivity analysis, we used the Partial Rank Correlation Coefficients (PRCC). These measures provide a measure of monotonicity, it takes values between -1 and 1 [?]. The lower end of the range

indicates strong evidence of a decreasing relationship between the given parameter and $\mathcal{R}_0$, while a value close to 1 presents strong evidence of a positive effect in $\mathcal{R}_0$. A sample size of 10,000 and uniform distributions in the parameter ranges were used to compute the PRCC.

Since the value of $\mathcal{R}_0$ depends on the venue and host behavior parameters, the PRCCs have been computed for each pair (λ, ρ_T) and plotted in a heat map depicted in Fig. 1. Our results confirm that increasing the decay rates (μ_F, μ_H) reduces the value of $\mathcal{R}_0$ while increasing pickup transfer efficacy (from fomites to hand) increases $\mathcal{R}_0$. These statements are valid throughout all the values of λ and ρ_T considered in this manuscript (plots not shown). Only the PRCC values of ϕ_H are depicted in Fig. 1. Red colors indicate an decreasing relationship with $\mathcal{R}_0$ and thus a positive effect to reduce the severity of the epidemic.

For influenza ϕ_H has a negative impact on $\mathcal{R}_0$, this means that increasing the value of ϕ_H can help mitigate the severity of the epidemic. This can certainly be due to the high decay rate in hands. In contrast, for the other three pathogens, the PRCCs differ depending on the values of ρ_T and λ. In a venue with high touching rates and high proportion of touchable surfaces (school/daycare) increasing the proportion of pathogens deposited in hands can be beneficial to reduce the severity of an epidemic. If a larger surface area is available for touching and pathogens spread uniformly in this medium the dilution helps prevent the spread of disease. If, however, the surface space is limited and touching rates are high (subway) reducing the value of ϕ_H predicts lower values of $\mathcal{R}_0$.

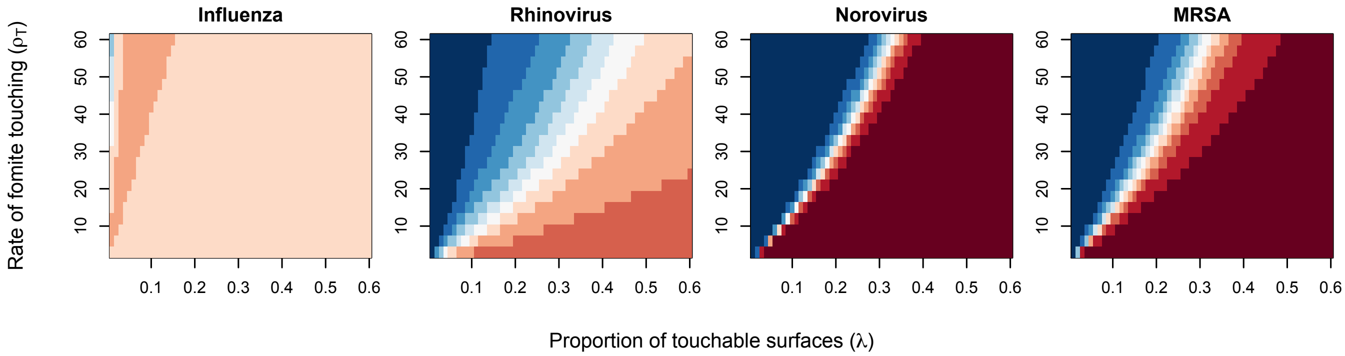

Figure 1: Heat map of the PRCC values of ϕ_H for each pair ρ_T and λ. Red colors indicate a decreasing relationship with $\mathcal{R}_0$, blue values indicate evidence for an increasing relationship. {PRCC}
