# Supplementary material for: Fomite-mediated transmission as a sufficient pathway: a comparative analysis across three viral pathogens
Source: BMC Infect Dis. 2018 Oct 29;18:540. doi: 10.1186/s12879-018-3425-x (PMC6206643; doi:10.1186/s12879-018-3425-x)
Supplement: Supplementary file 2 — Code Files. This file contains the code used for generating figures 1, 3, and 4. (DOCX 22 kb) [file 12879_2018_3425_MOESM2_ESM.docx]

#Code to generate figure 1

library(ggplot2)

x = c(1/88.2, 1/0.767, 1/1.07)

y = c(0.1,0.2,0.051)

names = c("Influenza", "Rhinovirus", "Norovirus")

png('Figure1a.png',width=6,height=6,units='in',res=300)

plot(x,y,pch=21,bg='blue',xlab="Persistence on Hands",ylab="Fomite-Hand Transfer Efficiency",cex.lab=1.5,cex.axis=1.5,cex=2)

xText = c(0.18, 1.1, 1/1.07)

yText = c(0.1025,0.2,0.065)

text(xText,yText,labels=names,cex=1.2)

dev.off()

#png('Figure1b.png',width=6,height=6,units='in',res=300)

#y1 = c(0.1,0.25,0.4,0.5)

#x1 = c(20,5,40,50)

#plot(1, type="n",xlab="Proportion of Accessible Surfaces",ylab="Touching Rate",xlim=c(0,0.75), ylim=c(0,60),cex.lab=1.5,cex.axis=1.5,cex=1.5)

#dev.off()

##Code to generate figure 3

# Creates level curves of R0 for the 4 pathogens

# all rates are given in hours

#library(Cairo)

####################################################################################

######################### Define the functions #########################

####################################################################################

R0 <- function(lamb, rhoT, param){ # this function computes R0 for a given set of parameters

with(as.list(param), {

kappa <- 0.0012*0.005/lamb

rhoFH <- rhoT*tauFH*kappa

rhoHF <- rhoT*tauHF

aF <- alpha*(1-phiH)*lamb

aH <- alpha*phiH

Pdep <- rhoHF/(muH+rhoHF+rho+thetaH)

Ppick <- (rhoFH*N) / ( rhoFH*N+muF+thetaF - ((rhoFH*N*rhoHF)/(muH+rhoHF+rho+thetaH)) )

Pinoc <- rho/(muH+rhoHF+rho+thetaH)

R0F <- Pinoc*Ppick*pi*(aF/gamma)

R0H <- Pinoc*Ppick*Pdep*pi*(aH/gamma)

R0F+R0H

})

}

R0F <- function(lamb, rhoT, param){ # this function computes R0 for a given set of parameters

with(as.list(param), {

kappa <- 0.0012*0.005/lamb

rhoFH <- rhoT*tauFH*kappa

rhoHF <- rhoT*tauHF

aF <- alpha*(1-phiH)*lamb

aH <- alpha*phiH

Pdep <- rhoHF/(muH+rhoHF+rho+thetaH)

Ppick <- (rhoFH*N) / ( rhoFH*N+muF+thetaF - ((rhoFH*N*rhoHF)/(muH+rhoHF+rho+thetaH)) )

Pinoc <- rho/(muH+rhoHF+rho+thetaH)

R0F <- Pinoc*Ppick*pi*(aF/gamma)

R0F

})

}

R0H <- function(lamb, rhoT, param){ # this function computes R0 for a given set of parameters

with(as.list(param), {

kappa <- 0.0012*0.005/lamb

rhoFH <- rhoT*tauFH*kappa

rhoHF <- rhoT*tauHF

aF <- alpha*(1-phiH)*lamb

aH <- alpha*phiH

Pdep <- rhoHF/(muH+rhoHF+rho+thetaH)

Ppick <- (rhoFH*N) / ( rhoFH*N+muF+thetaF - ((rhoFH*N*rhoHF)/(muH+rhoHF+rho+thetaH)) )

Pinoc <- rho/(muH+rhoHF+rho+thetaH)

R0H <- Pinoc*Ppick*Pdep*pi*(aH/gamma)

R0H

})

}

#####################################################################################

######################### Define the parameters #########################

#####################################################################################

N<-1000 ## global parameter

#lamb <- 0.005

#rhoT <- 60*0.1

######################### Influenza parameters #########################

param_Inf <- c( rho = 15.8,

muF = 0.121,

muH = 88.2,

tauFH = 0.1,

tauHF = 0.025,

phiH = 0.15,

gamma = 1/(6*24),

pi = 6.93e-05,

alpha = 1e4,

thetaF=0, thetaH=0 )

######################### Rhinovirus parameters #########################

param_Rhi <- c( rho = 15.8,

muF = 1.439,

muH = 0.767,

tauFH = 0.2,

tauHF = 0.2,

phiH = 0.15,

gamma = 1/(10.4*24),

pi = 2.46e-3,

alpha = 1e3,

thetaF=0, thetaH=0 )

######################### Norovirus parameters #########################

#Note: Decay on hands is biphasic. For now, I am calculating the total decay rate for the first hour since the R0

#calculation is based on hours.

param_Nor <- c( rho = 15.8,

muF = 0.288, #Original Nancy: 0.006, Exponential Alicia: 0.0154/24 - 6.91/24 -> 0.0006 - 0.288

muH = 1.07, #Original Nancy: 0.0001, Exponential Alicia: 0.0714*15 (0 decay after first 15 mins)

tauFH = 0.051,

tauHF = 0.094,

phiH = 0.5,

gamma = 1/(15*24),

pi = 4.78e-4,

alpha = 0.5*2.88*10^3,

thetaF=0, thetaH=0 )

####################################################################################

###################### Create matrices for level curves ######################

####################################################################################

x <- seq(0,0.6, by=0.01) ### range of values for lambda

y <- 60*seq(0, 1, by=0.05) ### range of values for rhoT

zR0_Inf <- outer(x, y, function(x,y) R0(x, y, param_Inf) )

zR0F_Inf <- outer(x, y, function(x,y) R0F(x, y, param_Inf) )

zR0H_Inf <- outer(x, y, function(x,y) R0H(x, y, param_Inf) )

zR0_Rhi <- outer(x, y, function(x,y) R0(x, y, param_Rhi) )

zR0F_Rhi <- outer(x, y, function(x,y) R0F(x, y, param_Rhi) )

zR0H_Rhi <- outer(x, y, function(x,y) R0H(x, y, param_Rhi) )

zR0_Nor <- outer(x, y, function(x,y) R0(x, y, param_Nor) )

zR0F_Nor <- outer(x, y, function(x,y) R0F(x, y, param_Nor) )

zR0H_Nor <- outer(x, y, function(x,y) R0H(x, y, param_Nor) )

#################################################################################

######################## Create level curves ########################

#################################################################################

#par(mfrow=c(1,1), mar=c(3,2.5,2,2), oma=c(1,1.4,0,0))

##### R0 #####

#contour(x,y,zR0_Rhi, xlab='', ylab='',, main=expression(paste('Rhinovirus ',R[0])))

#contour(x,y,zR0_Inf, nlevels=6, xlab='', ylab='', main=expression(paste('Influenza ',R[0])))

#contour(x,y,zR0_Nor, nlevels=5, xlab='', ylab='',, main=expression(paste('Norovirus ',R[0])))

#title(xlab=expression(paste("Proportion of touchable surfaces (", lambda, ")")),

# ylab=expression(paste("Rate of fomite touching (", rho[T], ")")), outer = TRUE, line = 0, cex.lab=1.3)

#################################################################################

#par(mfrow=c(1,1), mar=c(4,4,3,2))

##### R0 #####

#contour(x,y,zR0_Inf, nlevels=6, xlab='', ylab=expression(paste('Rate of fomite touching (', rho[T],', per hour)')), main=expression(paste('Influenza ',R[0])))

#contour(x,y,zR0_Rhi, xlab='', ylab='', main=expression(paste('Rhinovirus ',R[0])))

#contour(x,y,zR0_Nor, nlevels=5, xlab=expression(paste('Proportion of touchable surfaces (', lambda, ')')), ylab=expression(paste('Rate of fomite touching (', rho[T],', per hour)')), main=expression(paste('Norovirus ',R[0])))

#################################################################################

png('/Users/aliciakraay/Dropbox/Figure3Vomit.png',width=6,height=6,units='in',res=300)

par(mfrow=c(3,3), mar=c(2,2,3,1), oma=c(5.5,4.5,3,0))

#par(mfrow=c(3,3), mar=c(2,2,3,1) )

##### R0 #####

#par(mfrow=c(2,2), mar=c(3, 2.5, 2, 2))

contour(x,y,zR0_Inf, xlab='', ylab='', main=expression(bold(atop('Influenza',' '))))

contour(x,y,zR0_Rhi, xlab='', ylab='', main=expression(bold(atop("Rhinovirus",R['0']))))

contour(x,y,zR0_Nor, xlab='', ylab='', main=expression(bold(atop('Norovirus',' '))))

contour(x,y,zR0F_Inf, xlab='', ylab='', main='')

contour(x,y,zR0F_Rhi, xlab='', ylab='', main=expression(bold(R['0,F'])))

contour(x,y,zR0F_Nor, xlab='', ylab='', main='')

contour(x,y,zR0H_Inf, xlab='', ylab='', main='')

contour(x,y,zR0H_Rhi, xlab='', ylab='', main=expression(bold(R['0,H'])))

contour(x,y,zR0H_Nor, xlab='', ylab='', main='')

title(xlab=expression(paste("Proportion of accessible surfaces (", lambda, ")")),

ylab=expression(paste("Rate of fomite touching (", rho[T], ")")), outer=T, line = 1, cex.lab=1.5)

dev.off()

#Code to generate figure 4

# Creates level curves of R0 for the 4 pathogens

# all rates are given in hours

####################################################################################

######################### Define the functions #########################

####################################################################################

RatioRs <- function(lamb, rhoT, param){ # this function computes the ratio R_{C,F} / R_{0,F}

with(as.list(param), {

kappa <- 0.0012*0.005/lamb

rhoFH <- rhoT*tauFH*kappa

rhoHF <- rhoT*tauHF

aux1a <- ((rhoFH*N+muF)*(muH+rhoHF+rho)) - rhoFH*N*rhoHF

aux1b <- ((rhoFH*N+muF+thetaF)*(muH+rhoHF+rho+thetaH)) - rhoFH*N*rhoHF

aux1 <- aux1a/aux1b

aux2a <- (1-phiH)*lamb + (phiH*rhoHF/(muH+rhoHF+rho+thetaH))

aux2b <- (1-phiH)*lamb + (phiH*rhoHF/(muH+rhoHF+rho))

aux2 <- aux2a/aux2b

aux1*aux2

})

}

R0 <- function(lamb, rhoT, param){ # this function computes R0 for a given set of parameters

with(as.list(param), {

kappa <- 0.0012*0.005/lamb

rhoFH <- rhoT*tauFH*kappa

rhoHF <- rhoT*tauHF

aF <- alpha*(1-phiH)*lamb

aH <- alpha*phiH

Pdep <- rhoHF/(muH+rhoHF+rho+thetaH)

Ppick <- (rhoFH*N) / ( rhoFH*N+muF+thetaF - ((rhoFH*N*rhoHF)/(muH+rhoHF+rho+thetaH)) )

Pinoc <- rho/(muH+rhoHF+rho+thetaH)

R0F <- Pinoc*Ppick*pi*(aF/gamma)

R0H <- Pinoc*Ppick*Pdep*pi*(aH/gamma)

R0F+R0H

})

}

RatioF <- function(lamb, rhoT, param){ # this function computes the ratio R_{C,F} / R_{0,F}

with(as.list(param), {

kappa <- 0.0012*0.005/lamb

rhoFH <- rhoT*tauFH*kappa

rhoHF <- rhoT*tauHF

aux1 <- (rhoFH*N+muF)*(muH+rhoHF+rho) - rhoFH*N*rhoHF

aux2 <- (rhoFH*N+muF+thetaF)*(muH+rhoHF+rho+thetaH) - rhoFH*N*rhoHF

aux1/aux2

})

}

Grid_Rc_R0 <- function(param, ThetaF, qF, ThetaH, qH){ # Generates the grid for the level curve of R0 reductions

new_param <- param

new_param['thetaF'] <- qF*ThetaF

new_param['thetaH'] <- qH*ThetaH

x <- seq(0,0.3, by=0.01) ### range of values for lambda

y <- 60*seq(0, 1, by=0.05) ### range of values for rhoT

aux <- outer(x, y, function(x,y) RatioRs(x,y,new_param))

return(aux)

}

Grid_R0 <- function(param){ # Generates the grid for the level curve of R0

new_param <- param

new_param['thetaF'] <- 0

new_param['thetaH'] <- 0

x <- seq(0,0.3, by=0.01) ### range of values for lambda

y <- 60*seq(0, 1, by=0.05) ### range of values for rhoT

aux <- outer(x, y, function(x,y) R0(x,y,new_param))

return(aux)

}

GenGridRedF <- function(param, ThetaF, qF, ThetaH, qH){ # Generates the grid for the level curve of RF0/R0 reductions

new_param <- param

new_param['thetaF'] <- qF*ThetaF

new_param['thetaH'] <- qH*ThetaH

x <- seq(0,0.3, by=0.01) ### range of values for lambda

y <- 60*seq(0, 1, by=0.05) ### range of values for rhoT

aux <- outer(x, y, function(x,y) RatioF(x, y, new_param))

return(aux)

}

#####################################################################################

######################### Define the parameters #########################

#####################################################################################

N <- 1000

######################### Influenza parameters #########################

param_Inf <- c( rho = 15.8,

muF = 0.121,

muH = 88.2,

tauFH = 0.1,

tauHF = 0.025,

phiH = 0.15,

gamma = 1/(6*24),

pi = 6.93e-05,

alpha = 1e4,

thetaF=0, thetaH=0 )

######################### Rhinovirus parameters #########################

param_Rhi <- c( rho = 15.8,

muF = 1.439,

muH = 0.767,

tauFH = 0.2,

tauHF = 0.2,

phiH = 0.15,

gamma = 1/(10.4*24),

pi = 2.46e-3,

alpha = 1e3,

thetaF=0, thetaH=0 )

######################### Norovirus parameters #########################

#Note: Decay on hands is biphasic. For now, I am calculating the total decay rate for the first hour since the R0

#calculation is based on hours.

param_Nor <- c( rho = 15.8,

muF = 0.288, #Original Nancy: 0.006, Exponential Alicia: 0.0154/24 - 6.91/24 -> 0.0006 - 0.288

muH = 1.07, #Original Nancy: 0.0001, Exponential Alicia: 0.0714*15 (0 decay after first 15 mins)

tauFH = 0.051,

tauHF = 0.094,

phiH = 0.50,

gamma = 1/(15*24),

pi = 4.78e-4,

alpha = 2.88*10^3,

thetaF=0, thetaH=0 )

####################################################################################

###################### Create matrices for level curves ######################

####################################################################################

# Grid_Rc_R0(param, ThetaF, qF, ThetaH, qH)

# GenGridPropFtoR0(param, ThetaF, qF, ThetaH, qH)

zRR0_Inf <- array(0, dim=c(31,21,3)); zF_Inf <- array(0, dim=c(31,21,3))

zR0_Inf <- array(0, dim=c(31,21,3)); zF_Inf <- array(0, dim=c(31,21,3))

zRR0_Rhi <- array(0, dim=c(31,21,3)); zF_Rhi <- array(0, dim=c(31,21,3))

zR0_Rhi <- array(0, dim=c(31,21,3)); zF_Rhi <- array(0, dim=c(31,21,3))

zRR0_Nor <- array(0, dim=c(31,21,3)); zF_Nor <- array(0, dim=c(31,21,3))

zR0_Nor <- array(0, dim=c(31,21,3)); zF_Nor <- array(0, dim=c(31,21,3))

ThetaFs <- c(1/48,1/24, 1/12)

ThetaH <- 1/8

for (i in 1:3){

zRR0_Inf[,,i] <- Grid_Rc_R0(param_Inf, ThetaFs[i], 1, ThetaH, 1); #zRc_Inf[,,i] <- outer(x, y, function(x,y) RatioH(x, y, new_param))

zR0_Inf[,,i] <- Grid_R0(param_Inf);

zRR0_Rhi[,,i] <- Grid_Rc_R0(param_Rhi, ThetaFs[i], 1, ThetaH, 1); #zRc_Rhi[,,i] <- outer(x, y, function(x,y) RatioH(x, y, new_param))

zR0_Rhi[,,i] <- Grid_R0(param_Rhi);

zRR0_Nor[,,i] <- Grid_Rc_R0(param_Nor, ThetaFs[i], 1, ThetaH, 1); #zRc_Nor[,,i] <- outer(x, y, function(x,y) RatioH(x, y, new_param))

zR0_Nor[,,i] <- Grid_R0(param_Nor);

}

zR0C_Inf <- zRR0_Inf*zR0_Inf

zR0C_Rhi <- zRR0_Rhi*zR0_Rhi

zR0C_Nor <- zRR0_Nor*zR0_Nor

#################################################################################

######################## Create level curves ########################

#################################################################################

x <- seq(0,0.6, by=0.02) ### range of values for lambda

y <- 60*seq(0, 1, by=0.05) ### range of values for rhoT

png('Figure4.png',width=6,height=6,units='in',res=300)

par(mfrow=c(3,3), mar=c(2,2,3,1), oma=c(5.5,4.5,3,0))

image(x,y,zR0C_Inf[,,1]>1, col = terrain.colors(100), zlim=c(0,1), xlab='', ylab='', main=''); title(main=expression(bold(atop('Influenza',' '))))

#contour(x,y,zR0_Inf[,,1], levels=c(0.97,0.98), add=T)

contour(x,y,zRR0_Inf[,,1], nlevels=5, add=T)

image(x,y,zR0C_Rhi[,,1]>1, col = terrain.colors(100), zlim=c(0,1), xlab='', ylab='', main=''); title(main=expression(bold(atop("Rhinovirus","Cleaning 1/2-days"))))

#contour(x,y,zR0_Rhi[,,1], levels=c(0.8,0.9,0.95), add=T)

contour(x,y,zRR0_Rhi[,,1], nlevels=5, add=T)

image(x,y,zR0C_Nor[,,1]>1, col = terrain.colors(100), zlim=c(0,1), xlab='', ylab='', main=''); title(main=expression(bold(atop('Norovirus',' '))))

#contour(x,y,zR0_Nor[,,1], levels=c(0.5,0.7,0.9), add=T)

contour(x,y,zRR0_Nor[,,1], nlevels=5, add=T)

image(x,y,zR0C_Inf[,,2]>1, col = terrain.colors(100), zlim=c(0,1), xlab='', ylab='')

#contour(x,y,zR0_Inf[,,2], levels=c(0.94,0.95), add=T)

contour(x,y,zRR0_Inf[,,2], nlevels=5, add=T)

image(x,y,zR0C_Rhi[,,2]>1, col = terrain.colors(100), zlim=c(0,1), xlab='', ylab='',main='Cleaning 1/day')

#contour(x,y,zR0_Rhi[,,2], levels=c(0.6,0.8,0.95), add=T)

contour(x,y,zRR0_Rhi[,,2], nlevels=5, add=T)

image(x,y,zR0C_Nor[,,2]>1, col = terrain.colors(100), zlim=c(0,1), xlab='', ylab='')

#contour(x,y,zR0_Nor[,,2], levels=c(0.4,0.6,0.8), add=T)

contour(x,y,zRR0_Nor[,,2], nlevels=5, add=T)

image(x,y,zR0C_Inf[,,3]>1, col = terrain.colors(100), zlim=c(0,1), xlab='', ylab='')

#contour(x,y,zR0_Inf[,,3], levels=c(0.88,0.90,0.94), add=T)

contour(x,y,zRR0_Inf[,,3], nlevels=5, add=T)

image(x,y,zR0C_Rhi[,,3]>1, col = terrain.colors(100), zlim=c(0,1), xlab='', ylab='',main='Cleaning 2/day')

#contour(x,y,zR0_Rhi[,,3], levels=c(0.4,0.6,0.8,0.9), add=T)

contour(x,y,zRR0_Rhi[,,3], nlevels=5, add=T)

image(x,y,zR0C_Nor[,,3]>1, col = terrain.colors(100), zlim=c(0,1), xlab='', ylab='')

#contour(x,y,zR0_Nor[,,3], levels=c(0.2,0.4,0.6,0.8), add=T)

contour(x,y,zRR0_Nor[,,3], nlevels=5, add=T)

#mtext(paste('Hand cleaning per day:', ThetaH*24, '; ', ' Fomite decontamination per day:', ThetaFs[1]*24, ',', ThetaFs[2]*24, ',', ThetaFs[3]*24),

# side=3, col='black', line=1, outer=T, cex=1.2)

title(xlab=expression(paste("Proportion of accessible surfaces (", lambda, ")")),

ylab=expression(paste("Rate of fomite touching (", rho[T], ")")), outer=T, line = 1, cex.lab=1.5)

dev.off()

##############################################################################

##############################################################################

##### R0F / R0 #####

#par(mfrow=c(3,3), oma=c(0,1,2,0))

#image(x,y,zF_Inf[,,1], col = terrain.colors(100), zlim=c(0,1), xlab=expression(lambda), ylab=expression(rho[T]), main='\n Influenza' )

#contour(zF_Inf[,,1], nlevels=5, add=T)

#image(x,y,zF_Rhi[,,1], col = terrain.colors(100), zlim=c(0,1), xlab=expression(lambda), ylab=expression(rho[T]), main='\n Rhinovirus' )

#contour(zF_Rhi[,,1], nlevels=5, add=T)

#image(x,y,zF_Nor[,,1], col = terrain.colors(100), zlim=c(0,1), xlab=expression(lambda), ylab=expression(rho[T]), main='\n Norovirus' )

#contour(zF_Nor[,,1], nlevels=5, add=T)

#image(x,y,zF_Inf[,,2], col = terrain.colors(100), zlim=c(0,1), xlab=expression(lambda), ylab=expression(rho[T]))

#contour(x,y,zF_Inf[,,2], nlevels=5, add=T)

#image(x,y,zF_Rhi[,,2], col = terrain.colors(100), zlim=c(0,1), xlab=expression(lambda), ylab=expression(rho[T]))

#contour(x,y,zF_Rhi[,,2], nlevels=5, add=T)

#image(x,y,zF_Nor[,,2], col = terrain.colors(100), zlim=c(0,1), xlab=expression(lambda), ylab=expression(rho[T]))

#contour(x,y,zF_Nor[,,2], nlevels=5, add=T)

#image(x,y,zF_Inf[,,3], col = terrain.colors(100), zlim=c(0,1), xlab=expression(lambda), ylab=expression(rho[T]))

#contour(x,y,zF_Inf[,,3], nlevels=5, add=T)

#image(x,y,zF_Rhi[,,3], col = terrain.colors(100), zlim=c(0,1), xlab=expression(lambda), ylab=expression(rho[T]))

#contour(x,y,zF_Rhi[,,3], nlevels=5, add=T)

#image(x,y,zF_Nor[,,3], col = terrain.colors(100), zlim=c(0,1), xlab=expression(lambda), ylab=expression(rho[T]))

#contour(x,y,zF_Nor[,,3], nlevels=5, add=T)

#title(paste('Hand cleaning per day:', ThetaFs[1]*24, ',', ThetaFs[2]*24, ',', ThetaFs[3]*24, '; ', ' Fomite decontamination per day:', ThetaH*24), outer=TRUE)

#title('\n \n Reduction in RF', outer=TRUE)
